# Spatiotemporal Variation and Networks in the Mycobiome of the Wheat Canopy

**DOI:** 10.3389/fpls.2017.01357

**Published:** 2017-08-02

**Authors:** Rumakanta Sapkota, Lise N. Jørgensen, Mogens Nicolaisen

**Affiliations:** Department of Agroecology, Aarhus University Aarhus, Denmark

**Keywords:** mycobiome, wheat, *Zymoseptoria tritici*, *Cryptococcus*, *Sporobolomyces*, cultivar, pathogen, microbiome

## Abstract

The phyllosphere is an important habitat for a diverse microbiome and an important entry point for many pathogens. Factors that shape the phyllosphere microbiome and also the co-existence among members and how they affect disease development are largely understudied. In this study we examined the wheat mycobiome by using metabarcoding of the fungal ITS1 region. Leaf samples were taken from four cultivars grown at two locations in Denmark. Samples were taken from the three uppermost leaves and at three growth stages to better understand spatiotemporal variation of the mycobiome. Analysis of read abundances showed that geographical location had a major effect in shaping the mycobiome in the total dataset, but also leaf position, growth stage and cultivar were important drivers of fungal communities. Cultivar was most important in explaining variation in older leaves whereas location better explained the variation in younger leaves, suggesting that communities are shaped over time by the leaf environment. Network analysis revealed negative co-existence between *Zymoseptoria tritici* and the yeasts *Sporobolomyces, Dioszegia*, and *Cystofilobasidiaceae.* The relative abundance of *Z. tritici* and the yeasts was relatively constant between individual samples, suggesting that fast growing fungi rapidly occupy empty space in the phyllosphere.

## Introduction

Wheat (*Triticum aestivum*) is essential for global food production and is among the most commonly grown crops. Wheat production yields are highly reduced by a number of fungal leaf pathogens such as *Zymoseptoria tritici, Phaeosphaeria nodorum, Puccinia striiformis*, and *Blumeria graminis* f. sp. *tritici* (Oerke, 2006). Due to the economic importance, life cycles, epidemiology and control methods of these pathogens are well known. Plant pathogens and their disease progression have traditionally been regarded as a relationship between the plant, the pathogen and the environment (Agrios, 2005). This view, however, has failed to take into account the vast communities of commensal and mutualistic microorganisms, and other pathogens that colonize the plant host along with the pathogen itself, and their potential effects on disease development. It is our belief that a better understanding of pathogens in a phytobiome perspective may lead to other ways of controlling disease in a more sustainable way. However, due to the paucity of appropriate methods for studying microbiomes until recently, limited knowledge is available so far about phyllosphere microbial communities. Today novel metabarcoding technologies have fuelled microbial community studies enabling much more detailed insights into these communities and the factors that drive their structure.

An accumulating body of evidence suggests that microbial communities can negatively or positively affect the health and fitness of plants by promoting disease (Bertelsen et al., 2001), reducing pathogen infections through competition or antagonism (Saikkonen et al., 1998; O’Hanlon et al., 2012; Philippot et al., 2013; Nicolaisen et al., 2014) or by increasing tolerance to abiotic stresses such as heat or drought (Hardoim et al., 2008; Lau and Lennon, 2012). Sterile plants are more susceptible to infection (Innerebner et al., 2011; Ritpitakphong et al., 2016), demonstrating that microbial communities have an antagonistic effect against pathogens (Vorholt, 2012). Surprisingly, several studies have shown that fungicide treatments only have limited effect on the overall structure of fungal communities in wheat (Karlsson et al., 2014; Sapkota et al., 2015).

To date, limited information is available about how phyllosphere microbial communities vary across spatial and temporal scales, and how they are affected by host and environmental factors, and by agronomic manipulation. Likewise, little information is available about how members of the microbiota interact and how this interaction influences the development of diseases in plants. In a recent study we examined the phyllosphere mycobiome of small grain cereal crop species. We found that host genotype at species level but also at cultivar level was an important driver of fungal communities, and further we found that host specific pathogens live in a ‘sea’ of non-host specific fungi (Sapkota et al., 2015).

In the present study our objective was to study the local variation in fungal communities within the plant canopy at different growth stages and in different host backgrounds. Furthermore, we were interested in highlighting the interactions between fungal taxa in the phyllosphere, particularly in taxa that were interacting with important wheat pathogens. Our hypothesis was that fungal communities vary as a function of leaf age and host, and that single members of the community compete for space and nutrients. We investigated this by metabarcoding of the fungal internal transcribed region 1 (ITS1) from samples of leaves from four wheat cultivars grown at two locations in Denmark and by taking samples from leaf positions 1, 2, and 3 numbered from the head, at different growth stages.

## Materials and Methods

### Study Sites, Treatments and Plant Material

The study sites were located in Flakkebjerg and Horsens, Denmark (55.321547 N, 11.385 E and 55.8135 N, 9.9259 E, respectively). Both sites are experimental research fields located approximately 110 km apart.

Commercial cultivars of wheat (Hereford, Jensen, Mariboss and Tabasco, **Supplementary Table S1**) were sown in 2013 in 15 m^2^ plots at each location and in three replicates in a split-plot design. The plots were not treated with fungicides. Ten leaves from each of the leaf positions 1, 2, and 3 from the top were collected randomly in the plot, regardless of disease symptoms on the leaves, at growth stage [GS, according to Lancashire et al. (1991)] 49–51 (June 6, 2013) (only from Flakkebjerg), 69-71 (Flakkebjerg: June 28, 2013; Horsens: July 2, 2013) and 75–78 (Flakkebjerg: July 8, 2013; Horsens: July 11, 2013). The 10 leaves from each sampling were then pooled to constitute one sample.

Disease severity of septoria leaf blotch and powdery mildew was visually assessed by estimating the percentage of leaf coverage of specific symptoms according to Anonymous (2012) and noted as an average value of the three replicate plots (**Supplementary Table S1**).

### DNA Extraction, PCR Amplification and Pyrosequencing

Lyophilized pools of leaves were homogenized in liquid N_2_ with eight steel balls (0.5 mm) using a Geno/Grinder 2000 (OPS Diagnostics, Bridgewater, NJ, United States) at 1500 strokes/min. 100 mg of this was used for DNA extraction using a KingFisher^TM^ mL (Thermo Fisher Scientific Inc., Watham, MA, United States) with a sbeadex kit (LGC Genomics, Middlesex, United Kingdom) according to the manufacturer’s protocol.

Amplicon sequencing was done as described by Sapkota et al. (2015). In brief, we used the internal transcribed spacer 1 (ITS1) as a marker. To generate amplicons for sequencing, primers ITS1-F (Gardes and Bruns, 1993) and 58A2R (Martin and Rygiewicz, 2005) were used. Ten-nucleotide multiplex identifier (MID) primer tags were added to the forward primer (**Supplementary Table S1**). PCR reactions contained 1 × PCR reaction buffer, 1.5 mM MgCl_2_, 0.2 mM dNTPs, 1 μM each primer, 1 U of Taq DNA recombinant polymerase (Promega Corporation, Madison, WI, United States) and 1 μl (app. 5 ng) of DNA template in a final volume of 25 μl. All amplifications were conducted in a GeneAmp PCR System 9700 thermal cycler (Thermo Fisher Scientific) using 94°C for 5 min, followed by 35 cycles at 94°C for 30 s, 48°C for 30 s, 72°C for 1 min, and a final elongation step at 72°C for 10 min. Amplicons were pooled in equimolar amounts. The amplicons were resolved on 1.5% agarose gels, and a smear of amplicons at approximately 280–360 base pairs was excised from the gel and purified using a QIAquick Gel Extraction Kit (QIAGEN GmbH, Hilden, Germany). The sample pools were sequenced by Eurofins MWG on a GS Junior 454 Sequencer using titanium chemistry.

### Bioinformatics and Statistical Analysis

Sequence data processing was essentially as described (Sapkota et al., 2015). In brief, raw sequence files were converted into flowgrams and sequences were analyzed using QIIME v. 1.8 (Caporaso et al., 2010). Flowgrams were subjected to Amplicon noise to remove reads containing mismatching primers and MID sequences, PCR and sequencing errors, and chimeras (Quince et al., 2011). ITS1 sequences were extracted using ITSx extractor version 1.0.6 (Bengtsson-Palme et al., 2013). ITS1 reads were then clustered using the pick_open_reference_otus.py script at 97% similarity level in UClust (Edgar, 2010). The UNITE database version 6 was used as reference for operational taxonomic unit (OTU) picking and assigning taxonomy (Abarenkov et al., 2010; Kõljalg et al., 2013). For species identification, representative sequences from each OTU with at least 100 sequences in total were subjected to queries in Basic Local Alignment Search Tool (BLAST) at NCBI since the UNITE database was unable to assign all OTUs to species level. Singletons were removed before constructing OTU tables. Diversity analysis was carried out using the core_diversity_analyses.py script in QIIME (Caporaso et al., 2010). Non-phylogenetic diversity estimates using observed species for α diversity and Bray–Curtis for β diversity were calculated. Removal of samples with low numbers of reads and transformation of data to relative abundances was done using the “decostand” function from the vegan package in R (R Core Team, 2016). For the β diversity, samples having less than 500 reads were removed, resulting in removal of 10 samples. Bray-Curtis distance matrices were subjected to permutational multivariate analysis of variance (PERMANOVA) (Anderson, 2001), with a permutation number of 999, in order to compare fungal community composition in different categories using the *adonis* function from the vegan package in R (Oksanen et al., 2013).

Network analysis was performed to visualize correlations among OTUs. Firstly, OTUs present in at least 50% of samples, and leaf samples having at least 1000 reads were selected for correlation analysis. Secondly, the OTU table was rarified at a sequencing depth of 1000 reads per sample, and Spearman’s rank correlations were calculated at OTU level in R as described earlier (Williams et al., 2014). Thirdly, correlations that were highly significant (*P*-value < 0.001) and with Spearman’s rho >0.5 and <-0.5 were considered to be correlated and selected for network analysis. Only OTU pairs present in at least 40% of samples were considered. Networks were visualized using Cytoscape^1^.

Reads and metadata have been published in NCBI SRA with the accession number SRP11131. Scripts used for the analysis will be available upon request.

## Results

### Data Characteristics

Leaf samples were collected from 24 wheat plots (two locations, four cultivars and three replicates) at different growth stages and from different leaf positions, eight plots (Jensen, Flakkebjerg, GS 69–71) were sampled twice giving a total of 164 leaf samples. Metabarcoding of the fungal ITS1 region using DNA from these samples resulted in a total of 452,544 reads after quality control, with each sample containing 2759 reads (±1145) on average. The reads were clustered into 212 OTUs at 97% identity after exclusion of singletons (**Supplementary Table S1**).

### Fungal Community Composition

**FIGURE 1:**
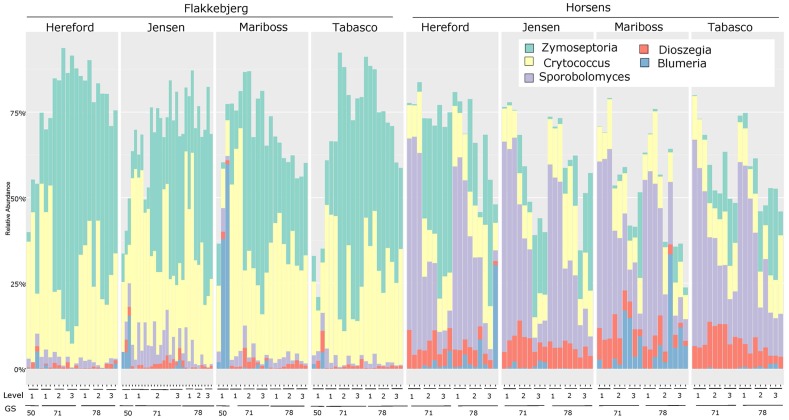
Bar chart showing the relative abundance of reads from the most dominant genera (*Zymoseptoria, Cryptococcus, Sporobolomyces, Dioszegia*, and *Blumeria*) for each cultivar at the two locations (Flakkebjerg and Horsens). Results are sorted according to location, cultivar, leaf position and growth stage. ‘50’ = growth stage 49–51. The three bars at each sampling point represent the three replicates.

**FIGURE 2:**
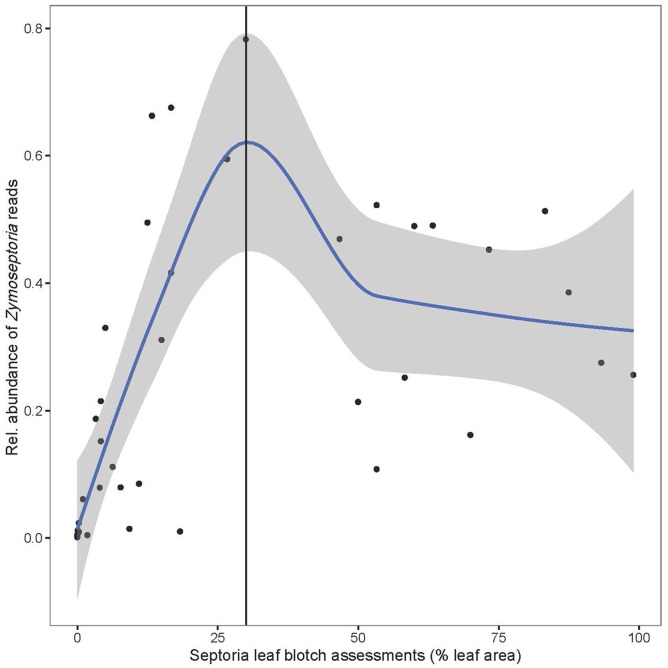
Average of relative abundance of *Zymoseptoria tritici* reads in the three replicate plots plotted vs. septoria leaf blotch assessments (% leaf area with symptoms as average of the three replicate plots). A 95 % confidence interval is shaded in gray. A vertical line indicates the approximate end of the linear relationship.

The fungal community structure was dominated by a few species as shown in **Figure 1**. Of the reads 99.3% could be assigned to 20 OTUs. Flakkebjerg plots were dominated by *Z. tritici* and *Cryptococcus* spp. and Horsens plots were dominated by *Z. tritici, Cryptococcus* spp., *Sporobolomyces* spp. and *Dioszegia* spp. A few scattered samples had a high abundance of *B. graminis* f. sp. *tritici* reads. Other taxa with a high relative abundance included Davidiellaceae, Pleosporales, Cystofilobasidiaceae, Tremellales, Phaeosphaeriaceae, Leucosporidiella, *Tilletiopsis* and *Monographella* (*Microdochium nivale*). The disease severity (percent leaf coverage) of septoria leaf blotch and powdery mildew were visually assessed in the plots and septoria leaf blotch assessments were compared to read abundance of *Z. tritici* (**Figure 2**). There was a close-to-linear relationship in samples with lower than 30% of the leaf area covered, whereas samples with a high disease severity did not show this linear relationship. The infection level of powdery mildew was too low for a meaningful comparison to relative read abundances of *B. graminis.*

### α-Diversity of Communities

A species accumulation curve indicated that the sampling depth was adequate as a plateau was approached (**Supplementary Figure S1**). Location had significant effects on fungal communities with the highest diversity in Horsens compared to Flakkebjerg (**Supplementary Figure S2**), whereas cultivar, leaf position and growth stage did not affect α-diversity significantly (data not shown).

### Geographical Location, Leaf Position, Cultivar and Growth Stage Affects Fungal Community Structures

**FIGURE 3:**
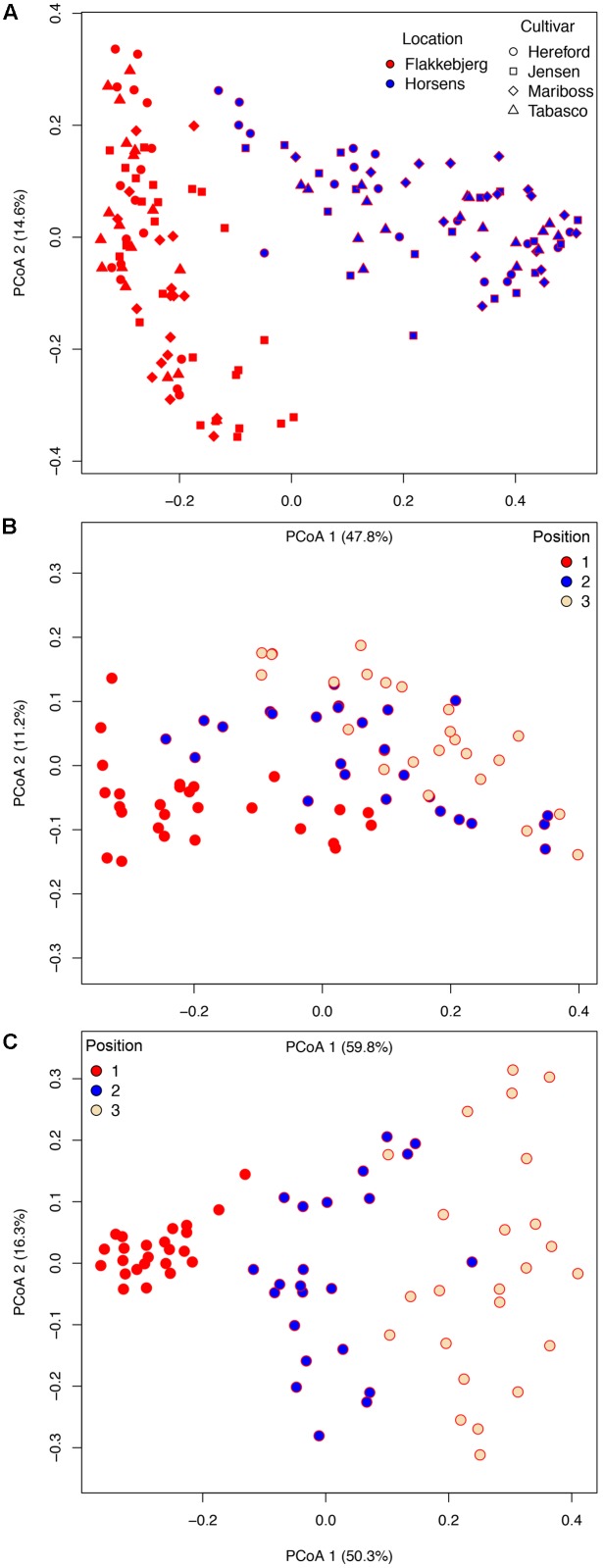
Bray–Curtis matrices visualized using principal coordinates analysis (PCoA) (axes 1 and 2) showing the distribution of samples according to **(A)** cultivar and location. In **(B,C)**, samples were split into the two locations, Flakkebjerg **(B)** and Horsens **(C)** and the distribution of samples according to leaf position is shown.

**FIGURE 4:**
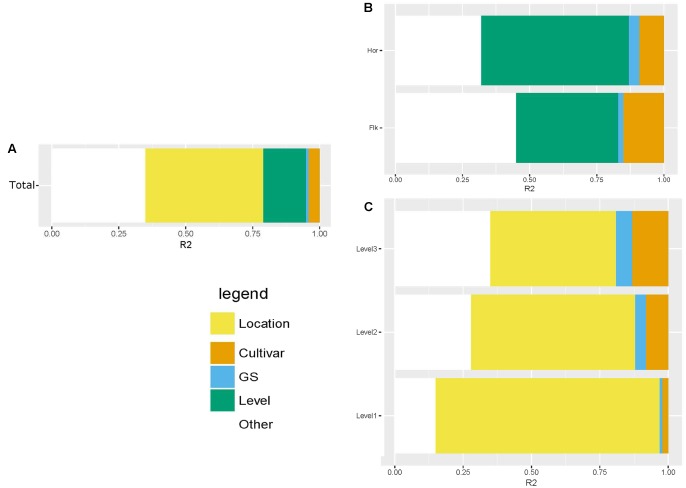
Adonis test showing the percent of variation explained by the different factors: location, leaf position, cultivar and growth stage. The figures shows the variation explained in the total dataset **(A)**, in the dataset split by location **(B)** and in the dataset split by leaf position **(C)**.

**FIGURE 5:**
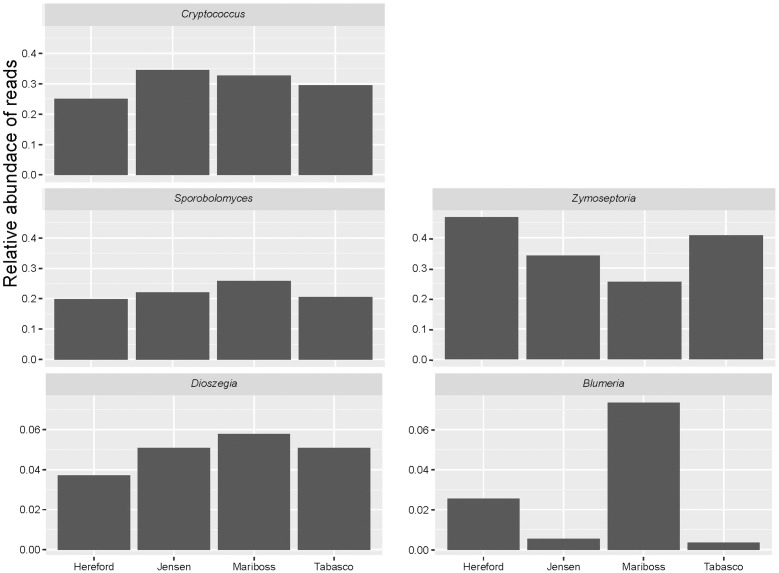
Relative abundance of reads assigned to the plant pathogens (*Zymoseptoria* and *Blumeria*) in the four different cultivars of wheat (Hereford, Jensen, Mariboss, and Tabasco), and of three yeasts that showed significant co-occurrence patterns with other fungi (*Dioszegia, Cryptococcus*, and *Sporobolomyces*). Note that the scale on the *Y*-axis is different between taxa.

We used a Bray–Curtis distance matrix to estimate β-diversity among samples. An unconstrained principal coordinate analysis (PCoA) demonstrated that most of the variation in the total dataset could be attributed to location (**Figure 3A**). The PCoA showed a strong separation of samples representing the communities from Flakkebjerg relative to the communities from Horsens, whereas cultivar did not have a notable effect on communities in the whole dataset. Leaf position separated samples in a PCoA plot, most remarkably in the Horsens plot (that was not heavily infected by *Z. tritici*) (**Figures 3B,C**). The observations from the PCoA were supported by adonis tests in which location was found to explain 44% of the variation of communities in the dataset (*R*^2^ = 0.44, *p* < 0.001). Leaf position explained 16% of the variation (*R*^2^ = 0.16, *p* < 0.001), whereas growth stage (*R*^2^ = 0.01, *p* < 0.001) and cultivar (*R*^2^ = 0.04, *p* < 0.001) only explained 1 and 4%, respectively, in the total dataset (**Supplementary Table S2**). By splitting the data into the two different locations and further into the three different leaf positions, cultivar had a much larger effect on fungal communities (**Figure 4**). It was observed that cultivar better explained variations in fungal communities on older leaves (position 3) compared to younger leaves (flag leaf), whereas geographical location was better in explaining variations in fungal communities on the younger leaves. Cultivar also had notable effects on individual pathogens and on some yeasts (**Figure 5**). *Z. tritici* reads were more abundant in Hereford and Tabasco and *B. graminis* reads were most abundant in Mariboss. The three yeasts (*Cryptococcus, Sporobolomyces*, and *Dioszegia*) shown in **Figure 5** were more equally distributed between cultivars, but showed the highest abundance in Jensen and Mariboss.

### Fungal Co-occurrence

**FIGURE 6:**
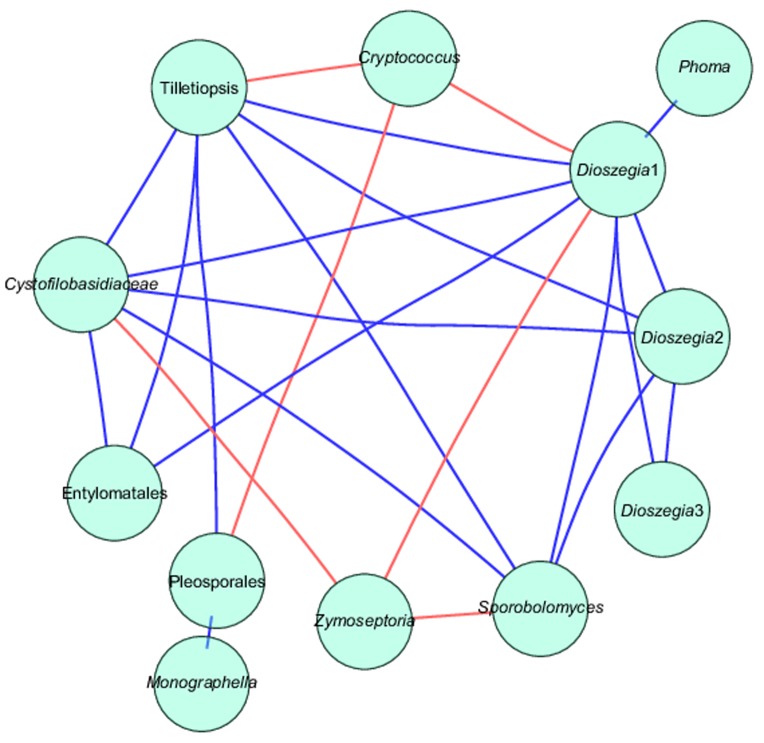
Network analysis of taxa showing significant positive or negative co-occurrences based on Spearman’s rank correlations. Blue lines represent positive correlations and red lines represent negative correlations. See also **Supplementary Table S2**.

*Zymoseptoria tritici* showed a highly significant negative co-occurrence with *Sporobolomyces, Cystofilobasidiaceae*, and *Dioszegia*, whereas *Cryptococcus* showed negative co-occurrence with Pleosporales, *Dioszegia* and *Tilletiopsis.* Several OTUs identified as *Dioszegia* spp. showed positive co-occurrence with *Tilletiopsis*, Entylomatales (smut fungi) and *Phoma*, whereas *Sporobolomyces* co-occurred positively with *Cystofilobasidiaceae, Dioszegia* and *Tilletiopsis* (**Figure 6** and **Supplementary Table S3**).

**FIGURE 7:**
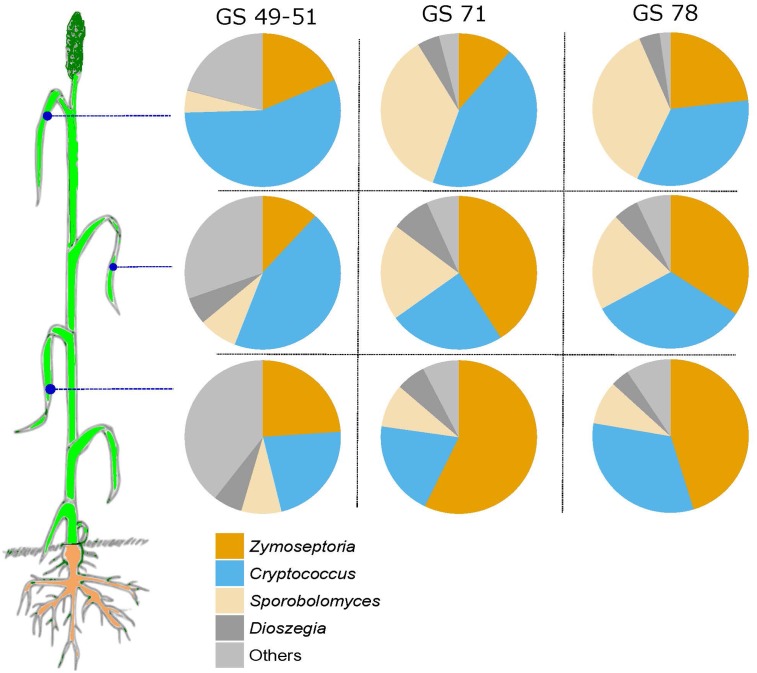
Pie-charts showing the relative abundance of sequence reads of *Zymoseptoria, Sporobolomyces, Cryptococcus, Dioszegia*, and ‘others’ in the three analyzed leaf positions and at growth stages 49–51, 69–71, and 75–78.

A comparison of read abundances of some of the most abundant taxa, *Z. tritici, Cryptococcus, Sporobolomyces*, and *Dioszegia*, revealed that the abundance of these taxa taken together was close to constant at the three different leaf positions but increasing from GS 49–51 to the later growth stages (**Figure 7**). *Z. tritici* was generally more abundant in the later growth stages and on older leaves (leaf position 3).

## Discussion

This study is a follow-up of a previous study: the first study focused on community differences at species level (winter- and spring barley, winter wheat, rye, triticale and oat) and found that there is a constant ‘sea’ of fungi across cereal phyllospheres with a few host specific pathogens and that host genotype is a major driver of microbial communities (Sapkota et al., 2015). The present study focuses on wheat and the effect of wheat cultivar, plant age and leaf position on the phyllosphere communities, and the co-existence networks formed within these communities among individual species, including pathogens.

Overall, 212 OTUs were identified in the dataset. This is lower than what is usually found in more complex environments such as soil, but comparable to what has been found in similar studies in the phyllosphere (Karlsson et al., 2014; Sapkota et al., 2015). Samples were dominated by a few OTUs but also hosting a number of rare taxa, as has also been observed in many other ecosystems (Magurran and Henderson, 2003). The role of these rare taxa for plant growth and productivity remains unclear, but some may be passive spore deposits from the air (Vorholt, 2012).

There was a remarkable difference in communities between locations. *Z. tritici* and *Cryptococcus* were abundant at both locations but were much more dominant in Flakkebjerg. In contrast, *Sporobolomyces* was dominant in Horsens and only occurred sporadically in Flakkebjerg, and *Dioszegia* was also much more abundant in the Horsens plots. The varying patterns were most likely caused by subtle differences such as different weather conditions, differences in crop rotations or other agronomical inputs at the two locations. However, the intention of this study was not to examine effects of these factors on the fungal populations in detail. *Blumeria* was present in most of the Horsens plots, but at varying amounts, whereas *Blumeria* was present in significant amounts in only a few plots in Flakkebjerg. *Blumeria* is forming large pustules on leaves and the sampling of only 10 leaves per plot may have resulted in stochastic effects in the case of low levels of pustules and thus high variation of read abundances between samples.

Principal coordinate analysis plots and adonis tests confirmed that location was the single factor tested that best explained variations in fungal communities in the total dataset. This could partly be an effect of the massive infection of *Z. tritici* in the Flakkebjerg plots. Previously, it was shown that specific pathogen attacks by their own right could have pronounced effects on phyllosphere communities (Reiter et al., 2002). The next most important factor shaping fungal communities was leaf position, whereas cultivar only had minor effects on the fungal communities in the total dataset. By splitting the data into location and further into the three leaf positions, we observed a much stronger effect of cultivar on the fungal communities. In our previous study (Sapkota et al., 2015), where we analyzed leaves from position 2, we found major effects of cultivar (explaining 29% of the variation in the wheat dataset), which was comparable to findings in the present study where we found that cultivar explained 30% of the variation of fungal communities in the leaves from position 2. Underlying this observation, there were remarkable effects of cultivar on individual taxa, including pathogens. Generally, our findings were in accordance with data from Danish official field trials^2^. These show that Mariboss is the most susceptible toward powdery mildew among the four cultivars used in this study, and Hereford is the most susceptible cultivar toward septoria leaf blotch. *Blumeria* read abundance was highest in Mariboss, and *Z. tritici* read abundance was highest in Hereford.

By splitting data based on leaf position, we observed that effects of cultivar on fungal communities increased from leaf position 1 (youngest leaf) to position 3, whereas location effects decreased. This could possibly be explained by the differences in leaf age at the three positions: in younger leaves fungal communities have not yet been fully shaped by the phyllosphere environment. This is supported by the observation that location has a relatively higher effect in younger leaves which, depending on the location (Horsens or Flakkebjerg), may have received different inocula from the air or from the soil. Comby et al. (2016) and Grudzinska-Sterno et al. (2016) also found variations in the composition of fungal communities depending on leaf age, and Grudzinska-Sterno suggested that more aggressive fungi dominated late in the season whereas many non-pathogenic fungi such as yeasts showed a clear decrease during the growth season. Another explanation could be differences in the spore dispersal mechanisms of the different fungi, for example, *Z. tritici* is a polycyclic pathogen that is spread by rain splashes from lower leaves to upper leaves, resulting in lower abundances on the flag leaf at earlier growth stages (Bockus et al., 2010). In a recent study, it was shown that leaf position (and age) had significant effects on the susceptibility of wheat to a biotrophic pathogen (wheat rust), the upper leaves being more susceptible, and it was suggested that the life strategy of the pathogen was a determining factor as for instance necrotrophic pathogens prefer older tissues (Farber and Mundt, 2017). These observations emphasize that samples should be taken from the same leaf position and age for mycobiome studies.

We compared read abundances of *Z. tritici* with visual assessments of septoria leaf blotch in the plots. At leaf coverages below 30% a linear relationship was observed, however, this relationship was not observed at higher rates of lesion coverage. This could be caused by the development of necrotic tissues that are not good growth media for *Z. tritici*. This observation of non-linearity could have implications for the assessment of cultivar resistance or fungicide effects as there seems to be no simple correlation between fungal biomass and visual symptoms at higher infection rates.

A network analysis revealed that *Z. tritici* co-occurs negatively with the yeasts *Dioszegia, Sporobolomyces*, and *Cystofilobasidiaceae*. A similar analysis of data from our previous study (Sapkota et al., 2015) and data from Karlsson et al. (2017) showed similar negative co-occurrence patterns between *Z. tritici* and several yeasts, thus supporting our results. The relative abundance of *Z. tritici* and the yeasts was strikingly constant, particularly across the samples from the three leaf positions, but also across cultivars and between GS 69–71 and 75–78. Read levels of *Sporobolomyces* were lower at GS 49–51 which is consistent with the fact that *Sporobolomyces* is known to progressively increase as the leaf matures (Fast, 1955). Whether the co-occurrence patterns observed between *Z. tritici* and the yeasts were a result of antagonism or whether their occurrence is a result of opposing requirements for growth factors remains unknown and would be an interesting subject for further study. *Sporobolomyces* has been shown to exhibit biocontrol effects against *Cochliobolus sativus* (Bashi and Fokkema, 1977), but it did not have any biocontrol activity against *Rhizoctonia cerealis* in the wheat stem base (Wachowska and Borowska, 2014). The potential of using yeasts as antagonists against wheat pathogens have been investigated previously. Grudzinska-Sterno et al. (2016) found negative correlations between *Cryptococcus* and *Z. tritici* in wheat, whereas Perello et al. (2002) did not observe any significant effects of *Cryptococcus* on spore germination in *Z. tritici.* In a recent study, *Dioszegia* was identified as a ‘hub’ species, mainly affecting phyllosphere bacteria (Agler et al., 2016). In the present study, several OTUs assigned to *Dioszegia* spp. showed highly significant both negative and positive co-occurrence patterns suggesting an important role of this genus in shaping not only bacterial but also fungal communities.

Studies of fungal communities in the phyllosphere are limited, without detailed observations on spatiotemporal variation. The present study not only describes the key fungal species in the wheat phyllosphere but also describes the local within-plant variation in communities over time, and the co-occurrence of fungal groups across locations, leaf position and cultivars. Our study demonstrates the complexity of fungal communities in the phyllosphere and provides valuable insights into the interactions among plant inhabiting organisms including pathogens.

## Conclusion

This report describes the wheat mycobiome and spatiotemporal variation. A network analysis visualized fungal co-occurrences in the wheat phyllosphere and demonstrated important negative and positive interactions, particularly with *Z. tritici*. It was shown that different mycobiomes exist on leaves at different positions and finally it was shown that mycobiomes are shaped by cultivar during leaf aging.

## Author Contributions

MN and LJ designed the experiments. RS and LJ performed the experiments. RS analyzed the data. RS, LJ, and MN drafted the manuscript. All authors read and approved the final manuscript.

## Conflict of Interest Statement

The authors declare that the research was conducted in the absence of any commercial or financial relationships that could be construed as a potential conflict of interest.

## Footnotes

1 http://www.cytoscape.org

2 https://www.landbrugsinfo.dk

## Supplementary Material

FIGURE S1 Species accumulation curve for the total dataset.

FIGURE S2 Boxplot showing Simpson (first plot) and Shannon (second plot) α-diversities of fungal communities at the two locations, Flakkebjerg and Horsens (LRO).

TABLE S1 Operational taxonomic unit (OTU) table including disease assessments of *Zymoseptoria tritici* and *Blumeria graminis* (percent coverage of leaf area). List of samples used in this study and their average scores for diseases based on visual assessments at the two locations. The total number of reads for each OTU and sample is shown.

TABLE S2 Adonis test carried out for partitioning of variance in fungal communities for the combined dataset and split data based on location and leaf level.

TABLE S3 Co-occurrence analysis of fungal OTUs. Spearman’s rank correlations were carried out at OTU level in R as described earlier (Williams et al., 2014). Correlations that were highly significant (*P*-value < 0.001) and with Spearman’s rho >0.5 and <-0.5 are listed.
