# Supplementary figures and images for: Spatiotemporal Variation and Networks in the Mycobiome of the Wheat Canopy
Source: Front Plant Sci. 2017 Aug 2;8:1357. doi: 10.3389/fpls.2017.01357 (PMC5539183; doi:10.3389/fpls.2017.01357)

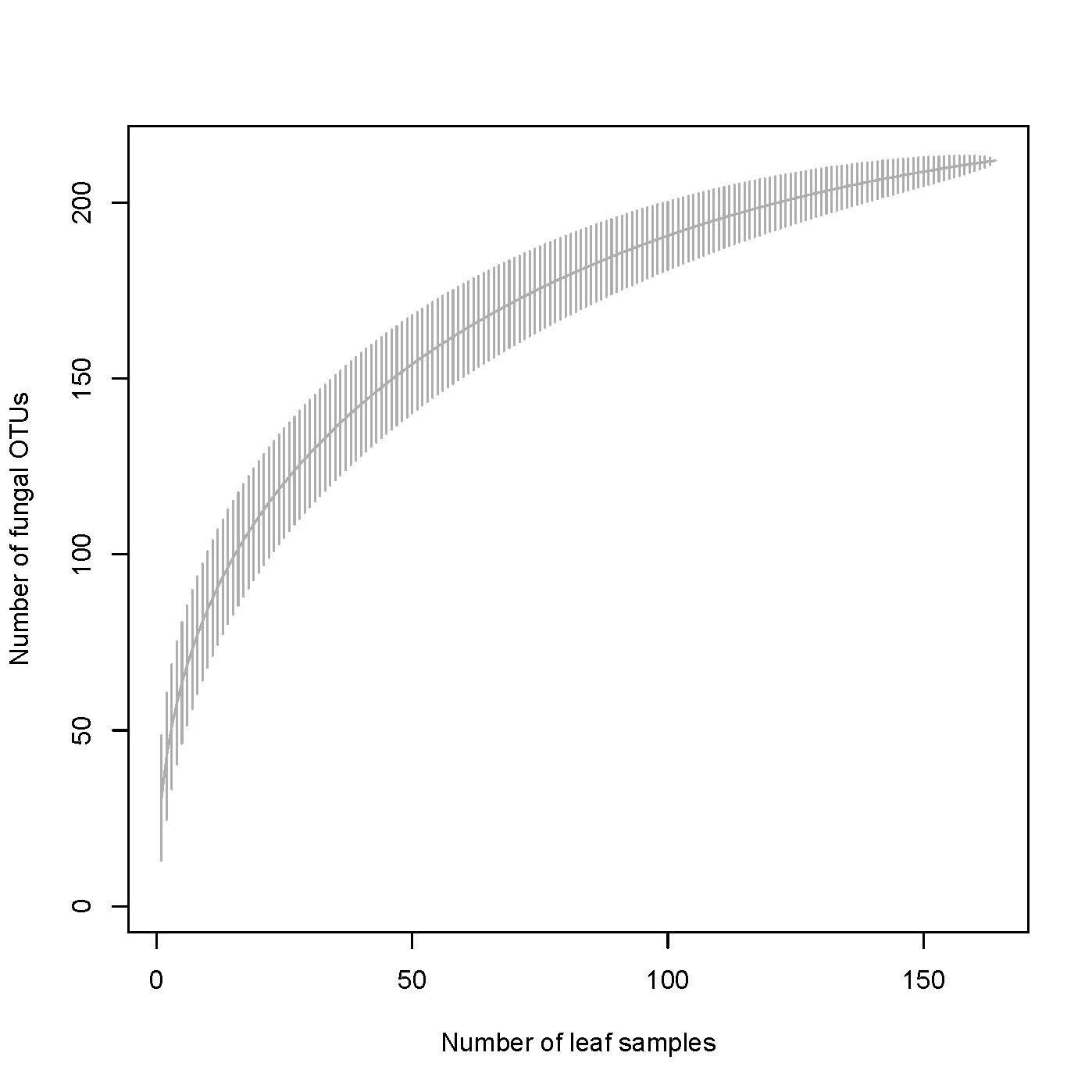

Supplement: FIGURE S1 — Species accumulation curve for the total dataset. [file Image_1.JPEG]

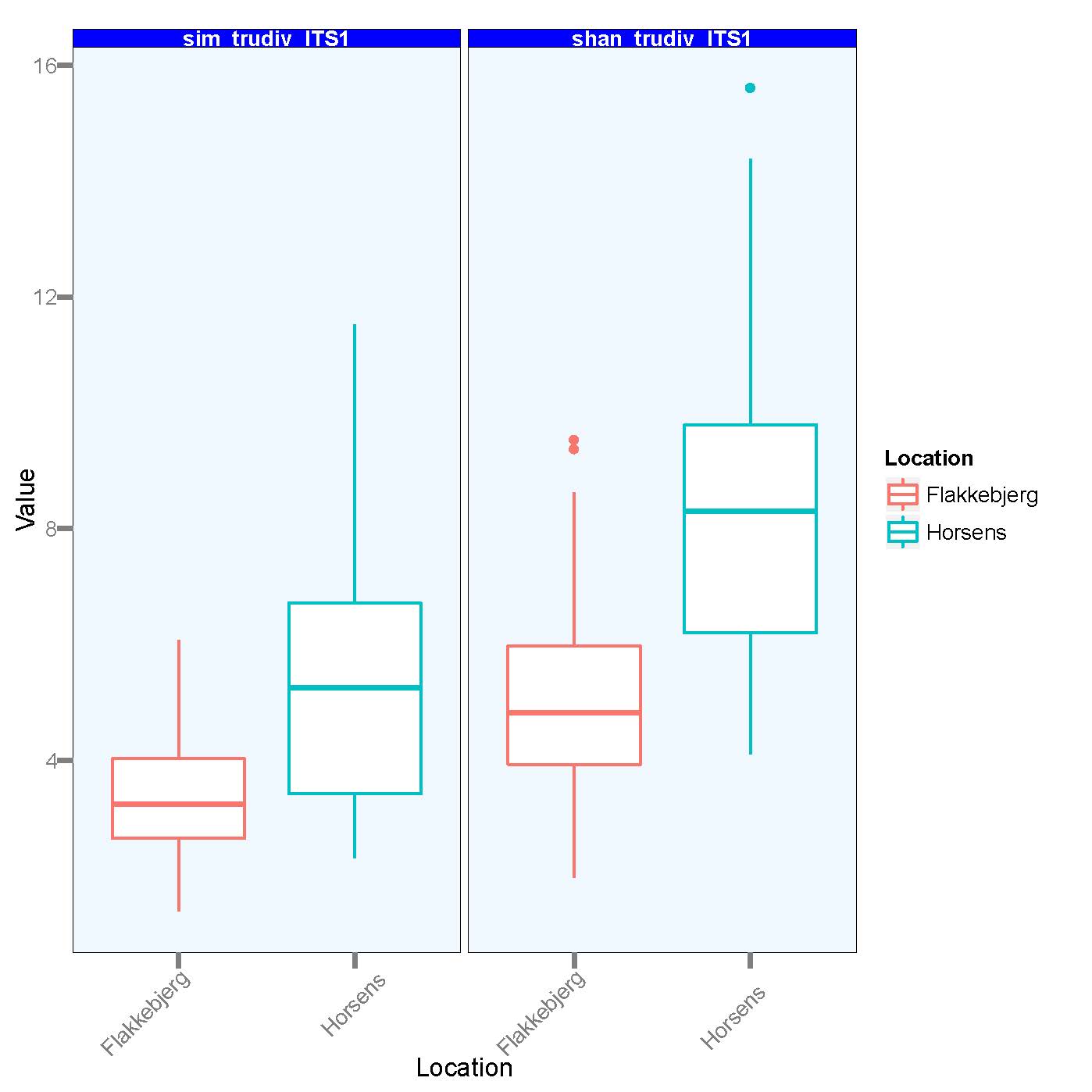

Supplement: FIGURE S2 — Boxplot showing Simpson (first plot) and Shannon (second plot) α-diversities of fungal communities at the two locations, Flakkebjerg and Horsens (LRO). [file Image_2.JPEG]
